# Sustained release of rhBMP-2 from microporous tricalciumphosphate using hydrogels as a carrier

**DOI:** 10.1186/s12896-016-0275-8

**Published:** 2016-05-20

**Authors:** Steffen Kissling, Michael Seidenstuecker, Ingo H. Pilz, Norbert P. Suedkamp, Hermann O. Mayr, Anke Bernstein

**Affiliations:** Center for Surgery, Department of Orthopedics and Trauma Surgery, Medical Center - University of Freiburg, Hugstetter Str.55, D-79106 Freiburg, Germany

**Keywords:** β-Tricalciumphosphate, Porous ceramics, Biomaterials, Alginate, Delayed drug release, Bone Morphogenetic Protein - 2 (BMP-2)

## Abstract

**Background:**

Tissue engineering and bone substitutes are subjects of intensive ongoing research. If the healing of bone fractures is delayed, osteoinductive materials that induce mesenchymal stem cells (MSCs) to form bone are necessary. The use of Bone Morphogenetic Protein - 2 is a common means to enhance effectiveness and accelerate the healing process. A delivery system that maintains and releases BMP biological activity in controlled fashion at the surgical site while preventing systemic diffusion (and thereby the risk of undesirable effects by controlling the amount of protein implanted) is essential.

In this study, we aimed to test a cylindrical TCP-scaffold (porosity ~ 40 %, mean pore size 5 μm, high interconnectivity) in comparison to BMP-2. Recombinant human BMP-2 was dissolved in different hydrogels as a carrier, namely gelatin and alginate cross-linked with CaCl_2_-solution, or a solution of GDL and CaCO_3_. FITC-labeled Protein A was used as a model substance for rhBMP-2 in the pre-trials. For loading, the samples were put in a flow chamber and sealed with silicone rings. Using a directional vacuum, the samples were loaded with the alginate-BMP-2-mixture and the loading success monitored by observing changes in a fluorescent dye (FITC labeled Protein A) under a fluorescence microscope. A fluorescence reader and ELISA were employed to measure the release. Efficacy was determined in cell culture experiments (MG63 cells) via Live-Dead-Assay, FACS, WST-1-Assay, pNPP alkaline phosphatase assay and confocal microscopy. For statistical analysis, we calculated the mean and standard deviation and carried out an analysis of variance.

**Results:**

Directional vacuum makes it possible to load nearly 100 % of the interconnected micropores with alginate mixed with rhBMP-2. Using alginate hardened with CaCl_2_ as a carrier, BMP-2's release can be decelerated significantly longer than with other hydrogels - eg, for over 28 days. The effects on osteoblast-like cells were an increase of the growth rate and expression of alkaline phosphatase while triggering no toxic effect.

**Conclusion:**

The rhBMP-2-loaded microporous TCP scaffolds possess proliferative and osteoinductive potential. Alginate helps to lower the local growth factor dose below the cytotoxic limit, and allows the release period to be lengthened by at least 28 days.

**Electronic supplementary material:**

The online version of this article (doi:10.1186/s12896-016-0275-8) contains supplementary material, which is available to authorized users.

## Background

The healing of bone fractures, especially critical size defects, poses a great challenge in medicine. Autologous bone grafts are routinely used, but they have drawbacks such as limited quantity and accessibility [1] and donor site morbidity. Alternative methods such as allografts, xenografts, and synthetic materials [2] were thus developed to repair bone. For human application, they must be non-toxic, non-carcinogenic, non-allergenic and non-inflammatory, as well as biocompatible and biofunctional. Many scaffolds made of synthetic materials have evolved from a variety of materials like metal, polymers, and ceramics.

Ceramics are defined as being inorganic, non-metallic materials generally characterized by extreme hardness, brittleness, low electrical and thermal conductivity, great compressive strength and high chemical resistance, the latter being demonstrated by their resistance to body fluids. Epple classifies ceramic biomaterials in two categories: hard bio-inert ceramics such as zirconia or alumina, and biodegradable ceramics [3]. The latter consist of mostly calcium phosphates, which are used in bone regeneration because of their similarity to inorganic bone components such as hydroxyapatite (HA). Many calcium phosphates are used in tissue engineering. Special attention should be paid to the β-Tricalciumphosphate (β-TCP) below. β-TCP has a slightly different composition and thus another calcium/phosphate ratio (HA 1.67; β-TCP 1.5) [4], resulting in more than twice HA’s solubility in water and better degradability than HA. In medicine, β-TCP is used alone or mixed with HA (biphasic calcium phosphate) as a bone substitute material [3]. For reasons of stability, the ceramic should degrade while the new bone is forming. β-TCP has proven to be a suitable material regarding its biodegradability and mechanical stability [5].

From a structural perspective, macroporous (100–1000 μm) scaffolds allow cell migration and boost angiogenesis [6] and resorption from the inner surface of the pores, while the microporosity within the scaffolds exerts a positive influence on bone integration in calcium phosphate scaffolds while resorption is taking place from the outer surface. There is evidence that bone density and its mass increase thereby, and that the scaffold’s osteointegration improves [7]. Combined macro- and microporous materials have recently demonstrated good ingrowth [8].

Since TCP is brittle and its high porosity leads to weak mechanical properties, our group postulated that >60 % dense TCP materials (porosity <40 %) could be used as scaffold for bone grafting and soft tissue fixation. We assumed that micropores (5 μm) would reduce brittleness by hindering crack propagation and preventing bone formation on the joint surface, and that their high interconnectivity would facilitate the nutrient transport essential for tissue engineering.

This concept was tested with a cylindrical TCP-scaffold (porosity nearly 40 %, mean pore size 5 μm, high interconnectivity, axial failure load 7200 N/cm ^2^) in vitro and vivo. The experiments included their use as fixation for ligamentoplasties, as bone substitution [9], and as a treatment for osteochondral defects in sheep [10, 11].

The implants displayed high mechanical stability (equivalent to healthy bone) [12]. The TCP resorption rate was 31 %, and 39 % of the resorbed TCP had been replaced by mineralized bone 24 weeks after implantation. Resorption took place almost exclusively on the surface, as expected [9]. This TCP scaffold seeded with chondrocytes is also suitable for treating osteochondral defects [11].

The repair of non-critically-sized bone defects can be achieved via osteoconductive TCP scaffolds. To repair critically-sized bone defects, osteoinductive materials inducing mesenchymal stem cells (MSCs) to form bone are necessary. The use of growth factors is a common means of enhancing effectiveness and accelerating the healing process: BMP-2 is one that triggers increased bone formation [13, 14]. What is essential is a delivery system that maintains and releases BMP biological activity in controlled fashion at the surgical site while preventing systemic diffusion (and thereby the risk of undesirable effects by controlling the amount of protein implanted). The combination of microporous calcium phosphate scaffolds and BMP-2 can accelerate healing up to 4-fold [15]. In view of these encouraging results, we aimed to test this scaffold’s function with Bone Morphogenetic Protein – 2 (BMP-2) in this study.

Two products using BMP-2 are commercially available and have been approved (InductOS®, Pfizer; InFUSE®, Medtronic) [16]. Both use collagen sponges and very high doses of BMP-2 (12 mg) without a system for retarded release. Clinically speaking, large doses of BMP-2 combined with a collagen matrix have triggered many side effects such as swelling [17], hematomas, and ossification at unintentional sites [18], and even a higher risk of cancer [19]. High in-vitro doses of BMP-2 have toxic effects on osteoblasts [20]. Another challenge when using BMP-2 for bone repair is its very short half-life lasting about 7 min [21]. We therefore need a delivery system that can prevent the growth factor from degrading prematurely and enable the local dose to be lowered while guaranteeing long-term delivery. Such a retarded therapeutic application should minimize complications.

The delivery can be retarded by different methods. You can bind substances adhesively or covalently to the bone substitute [22], load micro- and nanoparticles with agents [23], or use polymers like hydrogels or polylactides as a carrier [24].

The goal of this research was to achieve a pre-defined release of BMP-2 lasting several weeks by coupling the growth factor to a hydrogel and thus improve the mechanically-stable scaffold for bone replacement with enhanced osteoinductive properties.

## Methods

The ceramic we chose to examine is β-TCP having a total porosity of <40 %, while the interconnected micropores have an average pore diameter of 5 μm. This was used as cylindrically-shaped dowels 5 mm long and 7 mm in diameter, as described in previous studies [25].

### Production and characterization of the ß-TCP dowels

To manufacture the ß-TCP dowels, a paste containing 80 g of $\alpha$-tricalcium phosphate ($\alpha$-TCP; Ca_3_(PO_4_)_2_) and 20 g of tricalcium phosphate (Merck, Switzerland) was mixed for 2.5 min with 60.0 ± 0.2 g of a solution containing 0.2 M Na_2_HPO_4_ and +1 % polyacrylic acid (Fluka, Switzerland). During the hardening process (45 min), the paste was poured into plastic syringes whose tip had been cut off (Ø = 23 mm) and then covered with 10 ml of phosphate buffer saline pH 7.4 and incubated for 3 days at 60 °C. After drying, the ceramics were sintered at 1250 °C for 4 h, while heating and cooling took place at 1 °C/min. The cylinders were then machined to obtain plugs of 25 mm long and 7 mm in diameter and calcined at 900 °C to burn off all organic residues. Afterwards the plugs were cut into 5 mm long dowels and then washed in ethanol and distilled water in an ultrasonic bath to remove residual wear particles and sterilized by heat (200 °C, 4 h) for cell culture trials. This fabrication process has been described in greater detail in previous studies [5, 12, 25].

The fabricated ß-TCP plugs were phase pure as assessed by X-ray diffraction (XRD) and tested on a Bruker axs D8 Advance X-ray diffractometer (Billerica, USA).

The ceramics were microporous (mean pore size 5.05 ± 1.61 μm) with interconnecting pores and a total porosity of 35.2 ± 1.5 %. Porosity was assessed on a POROTEC Pascal 440 (Hofheim, Germany) mercury porosimeter.

### FITC-Protein A as a model substance

In the pretrial, Protein A (441 AS, 42 kDa) was used as a model substance for rhBMP-2 (396 AS, homodimer 30 kDa) because of its similar hydrodynamic volume and chemical structure. Apart from the financial aspects, Protein A can be FITC-labeled (Invitrogen, California), making measuring easy and precise.

### Hydrogels as carrier systems

We chose to evaluate three hydrogels: alginate 5 % w/v cross-linked with CaCl_2_ solution as an external Ca^2+^ source; self-hardening alginate [26] 5 % w/v cross-linked with 34 mM glucono-δ-lactone (GDL), obtained from Sigma-Aldrich (Missouri, USA), and 34 mM CaCO_3_ (Fluka, Switzerland); gelatin with a Bloom value of 300 (Gelita, Germany).

For the trials with rhBMP-2, alginate cross-linked with CaCl_2_ was chosen as a carrier because of its ideal release characteristics. The sodium alginate was produced as 5 % w/v alginate sol made with distilled water. To increase rhBMP-2 solubility, we added hydrochloric acid to pH 4. To ensure the best possible distribution in the sol, rhBMP-2 (Pfizer) [50 μg/ml] or FITC-labeled Protein A [50 μg/ml] were added to the distilled water immediately as production started. To prevent premature fading, the brine was homogenized in total darkness by wrapping the beaker glass in aluminum foil. To load the ceramics, we designed and manufactured a flow chamber from stainless steel. A directional vacuum was used for loading. There is a low vacuum of 40 mbar on one side of the flow chamber, while the pressure is atmospheric on the other side above the reservoir tank. In the flow chamber, the ceramic dowels are inserted in a silicone seal (FDA approved, LabMarket, Germany) that ensures that loading occurs only via the end surfaces of the microporous ceramic cylinders. This loading procedure guarantees a 100 % load of the micropores [27]. After loading, the dowels were incubated in 30 mM CaCl_2_ (Carl Roth, Germany) for 5 h at room temperature (ca. 20 °C) to harden the gel.

### Release kinetics

To simulate the physiological environment, the loaded dowels (FITC-Prot.A *n* = 7; BMP-2 *n* = 6) were incubated in phosphate buffer saline (gibco life technologies, California) at 37 °C and 5 % CO_2_. The phosphate buffer saline was completely replaced at each measuring point (1, 2, 3, 6, 9, 14, 21, 28 days).

The release of FITC-Protein A was measured via fluorescence spectroscopy (Tecan infinite 200) at 525 nm wavelength, while absorption was 495 nm wavelength. A six-point calibration curve was used (corr. R^2^ 0,977). The release of rhBMP-2 was measured by ELISA (R&D-Systems, Minnesota, USA) according to the protocol (seven-point Standard, corr. R^2^ 0,988).

### Osteoinductive capacity of released BMP-2

The efficacy of the released rhBMP-2 was calculated by its effect on osteoblast-like cells (MG63, ATCC CRL-1427). Those cells were grown in DMEM F12 Medium (Lonza, Switzerland) and incubated in 5 % CO_2_ at 37 °C.

#### Live-dead assay

First, the toxic and apoptotic potential of the amount of rhBMP-2 that had been released was assessed by fluorescence-activated cell sorting (FACS) with propidium iodide(1 mg/ml, Invitrogen, California). FACS is a specialized type of flow cytometry. It enables the analysis of cells that pass an electric voltage or a light beam at high speed. Depending on the cells’ shape, structure, and/or staining, different effects are produced from which the cells’ properties can be derived. Propidium iodide is discharged from vital cells only. Propidium iodide-positive cells represent dead or severely damaged cells.

1x10^4^MG63 cells were seeded per well on 24-well-plates and treated with 1 μg rhBMP-2 per 24 h for three days, which was the maximum daily release. Afterwards the cells were trypsinized, washed, and treated with 2 μl propidium iodide solution. The populations were detected by FACSCalibur flow cytometry (Becton Dickinson, New Jersey, USA). MG63-cells without rhBMP-2-treatment served as a control. 1000 events were counted per group.

To confirm our findings (3.3), we examined the cells on the surface of the scaffold using a live/dead Cell Staining Kit (Promokine, Germany). In this trial, 1x10^4^ MG63 cells were seeded on top of the scaffold and dyed with AM/EthD-III –dyeing after 24, 48 and 96 h.

Fluorescein dye has an excitation maximum at 495 nm and an emission maximum at 515 nm (green). Ethidium bromide is not absorbed by intact cell membranes, has an excitation maximum at 530 nm and an emission maximum at 635 nm (red). Vital cells reveal green, non-vital cells red fluorescence in fluorescence microscopy with blue light. The analysis was done manually.

#### Proliferation assay

Proliferation was measured with WST-1 Assay (Roche, Switzerland). For each measuring point at 24 h, 48 h, 96 h ß-TCP dowels (*n* = 2) were loaded with 5 % w/v alginate including 50 ng/ml rhBMP-2 and hardened with 30 mM CaCl_2_ as described above. The total amount of rhBMP-2 was 3.85 μg per plug. Dowels loaded only with 5 % w/v alginate served as control. 1x10^4 MG63-cells were seeded into 24-well-plates and incubated in 1 ml DMEM F12 medium. After a settle-time of 8 h, the loaded dowels were added to the medium and cells in cell culture inserts (BDFalcon, New Jersey, USA) with a pore size of 1 μm. At each measuring point the inserts and dowels were removed, 100 μl of WST-1-reagent was added and mixed well. After 2 h of incubation, absorption was measured at 450 nm wavelength (Tecan infinite 200, Tecan Group, Switzerland). Each sample was measured 4 times. The tetrazolium salts of the WST-1-reagent are cleaved to formazan by cellular enzymes. An increase in the number of viable cells raises the overall activity of mitochondrial dehydrogenases and thereby an increase in the total amount of formazan. This can be detected with a Reader at a wavelength between 420-480 nm.

#### Alkaline phosphatase assay and confocal microscopy

Osteoblastic differentiation was meted with SensoLyte® pNPP Alkaline Phosphatase Assay Kit (Anaspec EGT Group, California, USA). For each measuring point at 3d, 7d, and 14d, ß-TCP dowels (*n* = 3) were loaded with 5 % w/v alginate including 50 ng/ml rhBMP-2 and hardened with 30 mM CaCl_2_. The total amount of rhBMP-2 was 3.85 μg per dowel. Ceramics loaded only with 5 % w/v alginate served as a control. 1x10^4^ MG63-cells were seeded per well into 24-well-plates and incubated in 1 ml DMEM F12 Medium (Lonza, Switzerland). After 8 h of settle-time, the loaded dowels were added to the medium in cell culture inserts (BDFalcon, New Jersey, USA) as described above. At the measuring points the inserts and dowels were removed, the cells lysed with Triton X-100, and the supernatant collected for the assay. A seven-point calibration curve (corr. R^2^ 0,998) was set and each sample measured twice. The assay detects only biologically-active alkaline phosphatase. p-nitrophenyl phosphate is dephosphorylized by AP while the substrate turns yellow and can be detected at 405 nm wavelength with a Reader.

Another way to demonstrate AP increase is via confocal microscopy (Leica TCS SP2 AOBS spectral confocal microscope), where light can be separated from the object’s different levels. The use of fluorescent dyes and the observation laser’s different wavelengths enable assessment of the spatial distribution of labeled compounds. In this case, 1x10^4^ MG63-cells were seeded on Thermanox® (Thermo Scientific, Massachusetts, USA) membranes in 24-well-plates and incubated in 1 ml DMEM F12 medium and treated with rhBMP-2, as released from the scaffolds for 5 days. After the treatment, the cells were washed with phosphate buffer saline, fixed with methanol, protein-blocked and incubated with primary antibody (rabbit anti-alkaline phosphatase 1:100, Abcam, England) over night. The next day the membranes were washed again and incubated with secondary antibody (Donkey anti-Rabbit IgG, Alexa Fluor® 555; life technologies, California) for 1 h. Cell nuclei were dyed with DAPI (life-technologies, California).

### Statistics

Data is expressed as mean values ± standard deviation of the mean and analyzed by one-way analysis of variance (ANOVA). The level of statistical significance was set as *p* < 0.05. For statistical calculations Origin 9.1 Professional SR1 (OriginLab) was used.

## Results

### The most retardant hydrogel

FITC -Protein A release was very rapid from the gelatin and self-hardening alginate: 80.9 % of the gelatin and 76.1 % from the self-hardening alginate had been released after 24 h. After 9 days, the concentration was already under the detection limit.

The release of alginate with an external Ca^2+^ source measured only 35.6 % of the total after 24 h. After the release experiment, the scaffolds were broken and scrutinized under a fluorescence microscope. There was still some FITC Protein A left in the scaffolds loaded with alginate hardened with an external Ca^2+^ source, while there was nothing in the dowels loaded with self-hardening alginate and gelatin.

**Fig. 1:**
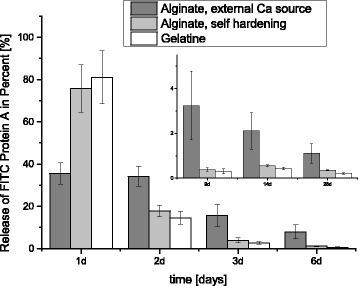
Comparison of the release kinetics of three different hydrogels regarding FITC Protein A measured by fluorescence reader in a 28-day-trial (incubation at 37 °C, Phosphate buffer saline changed completely at each measuring point): The alginate with external Ca2+ source reveals a significantly reduced burst release, while the second release phase is prolonged

**Fig. 2:**
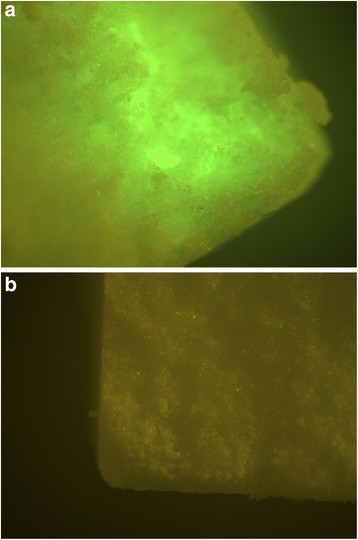
Fluorescence microscopy (Olympus BX-51, blue light filter) of alginate and FITC-Protein A-loaded dowels after 28 days’ incubation at 37 °C, Phosphate buffer saline changed completely at each measuring point; **a** (*left*): Remaining FITC Protein A in dowel (Alginate with external Ca2+ source) **b** (*right*): No FITC Protein A *left* in dowel loaded with self-hardening alginate

Thus the initial burst phase was significantly reduced while the second release phase was prolonged for over 28 days (cf. Fig. 1). After 28 days of release, there is still some FITC Protein A incorporated in micropores of the scaffold loaded with alginate hardened with an external Ca^2+^ source. In contrast to the self-hardening alginate, the incorporated FITC Protein A can be visualized by fluorescence microscopy (cf. Fig. 2a and b).

**Fig. 3:**
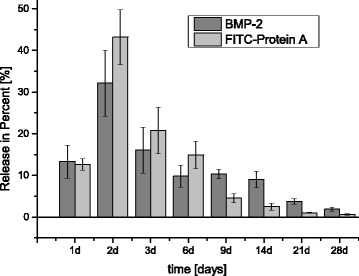
Considerably retarded release of BMP-2 from the externally-hardened alginate-loaded ß-TCP dowel in a 28-day-trial; BMP-2 load 3.85 μg/scaffold; incubation at 37 °C; Phosphate buffer saline changed completely at each measuring point

**Fig. 4:**
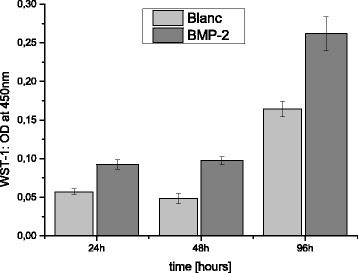
Significant proliferation differences in WST-1-Assay of MG63 cells after cultivation with BMP-2 and alginate-loaded ß-TCP scaffolds for 24 h, 48 h and 96 h

**Fig. 5:**
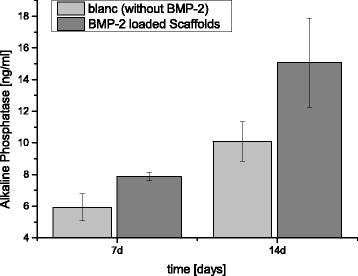
Expression of alkaline phosphatase in pNPP alkaline phosphatase assay after cultivation with BMP-2 and alginate-loaded ß-TCP scaffolds

### Retarded release of BMP-2 from an alginate-loaded ß-TCP dowel

Release of rhBMP-2 from the ceramics was very similar. Only the release on day 1 was lower, which can be ascribed to rhBMP-2 or FITC-Prot. A missing in the hardening-solution (CaCl_2_) and diffusion during the 5-h process.

Maximum release was 959 ± 222 ng/ml rhBMP-2 on the second day. After 14, 21 and 28 days, we still observed a release of 268 ± 59 ng/ml, 111 ± 20 ng/ml and 56 ± 16 ng/ml.

There is thus an initial burst phase followed by lengthier release phase lasting several weeks (cf. Fig. 3, raw data cf. Additional File 1).

### Proliferation and osteogenic potential

The results of FACS measuring with propidium iodide revealed no significant difference between the blanc and the group treated with 1 μg/ml rhBMP-2 (Table 1).

**Table 1:** MG63 cells after treatment with 1 μg BMP-2 per 24 h for 3 days display no differences in survival, measured by FACS with propidium Iodide

Group alive [%] dead [%]	
Blanc 97.6 ± 0.4 2.4 ± 0.4	
BMP-2 (1 μg/d) 97.8 ± 0.2 2.2 ± 0.2	

Results of the live/dead assay with AM/EthD-III staining displayed no significant difference between the scaffolds with and without loading with rhBMP-2.

In the WST-1-Assay, the groups displayed significant differences in proliferation rate at a significance level of *p* < 0.05. Proliferation of the MG63 cells incubated with the rhBMP-2 loaded scaffolds was significantly higher at all measured time points (cf. Fig. 4, raw data cf. Additional File 2). The increase was +38.3 % after 24 h, rising to +50.3 % after 48 h and only +35.4 % when reaching the culture plateau phase after 4 days.

The pNPP alkaline phosphatase assay revealed differences between the blancs and rhBMP-2-group (cf. Fig. 5, raw data cf. Additional File 3). The concentration of active AP increased from 5.9 ± 0.9 ng/ml to 7.9 ± 0.3 ng/ml, equaling 24.8 % after 7 days. The increase after 14d was from 10.1 ± 1.3 ng/ml to 15.1 ± 2.8 ng/ml, or 33 %. These results were not significant at a significance level of *p* < 0.05.

## Discussion

Bone morphogenetic proteins have been investigated intensively on both experimentally and clinically in orthopedic surgery. Especially in the early stages of bone regeneration, BMP-2 recruits local sources of skeletal progenitor cells and determines their course towards the osteogenic lineage. The current therapeutic application of BMP-2 for bone repair is nevertheless associated with unacceptable side effects and very high doses. The ideal carrier matrix should provide localized, retarded, and active BMP-2 delivery. Moreover, it should fulfill cell, attachment, proliferation, and differentiation requirements. We assumed that BMP-2 loaded microporous ß-TCP scaffolds, prepared by mixing BMP-2 solution with alginate and by loading the porous ceramics and hardening the alginate sol to a gel, would deliver a prolonged release system complying with these requirements. With this method, it is possible to include any concentration of one or more active ingredients in the scaffold. After the alginate has been hardened with calcium chloride, the diffusion from the gel -containing micropores into the surrounding area is delayed. Significant delays are observed in the release of antibiotics and growth factors when applying this method [24]. The amount of delay can be adjusted by altering the carrier substance's rheological properties. Alginate and calcium concentrations can therefore be modified. Alternatively, an alginate with longer molecular chains could be used. However, microporous ceramics cannot be loaded with fluids that are too viscous. The Bernstein working group has explored the limits of this loading process.

There are other means of controlling a substance’s release from bone-replacement materials. Zurlinden et al. demonstrated that you can bind rhBMP-2 covalently to calcium phosphate granular materials and delay the release up to a few hundred days by using sodium acetate (half-life up to 115 days) [28]. The amount of substances released daily decreases with the prolongation of desorption. The release period is longer than with hydrogels, but the amount of substances released daily is lower [29].

We have observed a burst release within the first 48 h of release (45.4 %), while 50.9 % of the BMP-2 release occurs during the remaining 28 days when a hydrogel is combined with a ß-TCP ceramic. The relatively fast release of BMP-2 may be attributed to the relatively large amount of BMP-2 adsorbed on hydrogel on the top of ceramic cylinders due to the ceramic loading process. At the last measuring point on day 28, 56 ± 16 ng/ml remained: about 2 % of the BMP-2 measured. This continuous BMP-2 release implies that abundant BMP-2 had been packed into the ceramic micropores. We noted homogeneous distribution of the hydrogel inside the entire ceramic. This amount suffices to stimulate osteoblasts’ migration and differentiation. In-vitro effects of BMPs are observed at very low doses (5-20 ng/mL) [16]. Most other studies have reported that substrate loaded with BMP-2 has a significantly fast in-vitro release rate. This has also revealed an initial burst release exceeding 50 %, and a slow slow-release stage [30, 31].

Many studies investigating BMP-2 delivery for bone repair have applied relatively high doses ranging from tens to hundreds of micrograms [32–34] and even 6-12 milligrams [35]. This makes treatment costly while exacerbating the drawback of side effects. High doses have also displayed negative effects on different bone cells in-vitro, inducing apoptosis from a few hundred nanograms upwards [36]. Our working group’s pretrials have yielded similar results.

Other recent studies have investigated the use of lower doses in the range of a few micrograms [37] achieving for example a 55 % increase in bone volume compared to empty defects 6 weeks after the implantation of polymer scaffolds containing only 1 μg BMP-2 in mice [38]. Findings like these encouraged us to reduce the total BMP-2 load down to 3.85 μg per scaffold, enabling us to reduce the daily release to significantly under the toxic and apoptotic threshold while ensuring a maximum release of 1 μg/day using the stiffest alginate from an external Ca^2+^ source (in comparison to a self-hardening alginate). The combination of an alginate from an external Ca^2+^ source with microporous ß-TCP ceramic provides a promising delivery system for BMP-2. There would be enough BMP-2 in-vivo in the first 4 weeks of bone healing, with just a little left for the remainder of the degradation period (52 weeks in a sheep model [11]). The other hydrogels we investigated (self-hardening alginate and gelatin) displayed a significant burst release due to their greater physical solubility. They are therefore unsuitable in a carrier system. Apart from the fact that the release of rhBMP-2 and Protein A is very similar, it is a good and inexpensive model substance.

BMP-2's in-vivo effects on osteoblasts and bone are well known [39]. Our trial was designed to demonstrate that released rhBMP-2 remains active after being dissolved in alginate following the loading procedure and release. We hypothesized that proliferation and the expression of osteogenic markers like AP would rise, as described in other studies [40].

BMP-2 proliferation seems to have contradictory effects on different types of cells. Fromigue’ et. al. postulated in 1998 that BMP-2 decreases proliferation up to 35 % in human bone marrow cells representing early progenitors [41] while other groups were unable to detect significant differences in osteosarcoma cell lines representing osteoblast-like-cells [42]. Having observed no other differences between the groups, we attribute this increase in proliferation to the released BMP-2.

**Fig. 6:**
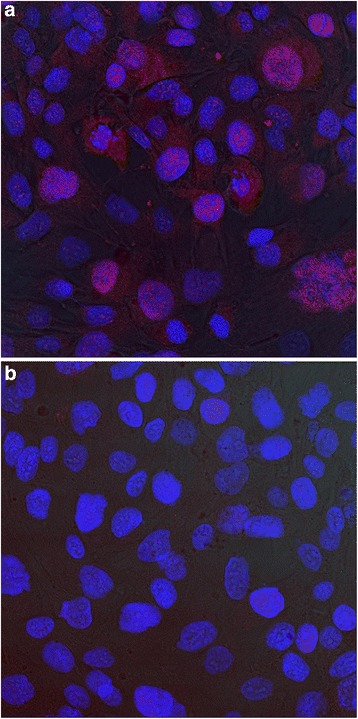
A (BMP-2) and 6 B (blanc): Confocal microscopy of MG63 cells after treatment with rhBMP-2 (*left*) and without (*right*) for 5 days, differences in expression of alkaline phosphatase. Cell nuclei *blue* (DAPI), AP *red* (rabbit anti-AP-AB; donkey anti rabbit, Alexa555)

The rhBMP-2 treated groups displayed higher AP activity than the groups lacking rhBMP-2 (cf. Fig. 6) (up to 33 % in our experiments), but not to a significant degree in the alkaline phosphatase assay. Perhaps the measuring points play a role; Draenert et. al. describe the maximum difference at 24 h using human osteoblasts [40]. Higher case numbers could also have helped achieve more convincing findings, as other working groups have employed [43]. Furthermore, rhBMP-2 handling could be optimized. A combination with heparin may lead to further improvement because heparin is a highly sulfated glycosaminoglycan known to bind to BMP-2 and stimulate its activity by protecting it from enzymatic degradation and the antagonistic actions of noggin [44], a protein involved in the development of tissues such as nerves, muscles, and especially bones. The same working group showed that acidic pH and the use of glass vials can lead to improved BMP-2 stability when compared with physiological pH and the use of plastic vials [45].

## Conclusion

It was our aim to amalgamate an osteoconductive, microporous, interconnective ß-TCP-scaffold with rhBMP-2. This scaffold consisting of a microporous ß-TCP ceramic and an alginate hydrogel with rhBMP-2 represents a promising delivery system for BMP-2.

We observed that BMP-2 exerts effects on osteoblast-like cells at very low doses over a lengthy period. We also believe that such scaffolds can lead to better and safer outcomes in humans.

## Additional files

Additional file 1: Dataset supporting Fig. 3. (TXT 3 kb) Additional file 2: Dataset supporting Fig. 4. (TXT 1 kb) Additional file 3: Dataset supporting Fig. 5. (TXT 1 kb)

## Abbreviations

AP: alkaline phosphatase
BMP: bone morphogenetic protein
Ca: calcium
CaP: calciumphosphate
Cl: chloride
DMEM F12: Dulbeccos modified eagle medium: nutrient mixture F12
ESEM: environmental scanning electron microscope
FACS: fluorescence-activated cell sorting
FCS: fetal calf serum
FITC: fluorescein isothiocyanate
GDL: glucono delta lactone
HA: hydroxyapatite
MG-63: immortalized human osteosarcoma cell line
MSC: mesenchymal stem cell
TCP: tricalciumphosphate
w/v: weight per volume
XRD: X-ray diffraction
